# Demographic and Clinicopathological Factors as Predictors of Lymph Node Metastasis in Merkel Cell Carcinoma: A Population-Based Analysis

**DOI:** 10.3390/jcm12051813

**Published:** 2023-02-24

**Authors:** Matteo Scampa, Daniel F. Kalbermatten, Carlo M. Oranges

**Affiliations:** Department of Plastic, Reconstructive, and Aesthetic Surgery, Geneva University Hospitals, University of Geneva, 1205 Geneva, Switzerland

**Keywords:** Merkel cell carcinoma, lymph node, sentinel lymph node biopsy, lymphadenectomy, epidemiology

## Abstract

Merkel cell carcinoma is an aggressive malignant skin tumor with high recurrence and low survival. Lymph nodal metastases are associated with a worse overall prognosis. Our aim was to assess how lymph node procedures and positivity are influenced by demographic, tumor, and treatment characteristics. The Surveillance, Epidemiology and End Results database was searched for all cases of Merkel cell carcinoma of the skin between 2000 and 2019. Univariable analysis was conducted using the chi-squared test with the aim of identifying differences in lymph node procedures and lymph node positivity for each variable. We identified 9182 patients, of which 3139 had sentinel lymph node biopsy/sampling, and 1072 had therapeutic lymph node dissection. Increasing age, increasing tumor size, and truncal location were associated with higher positive lymph node rates.

## 1. Introduction

Merkel cell carcinoma (MCC) is a rare skin tumor with neuro-endocrine features that affects mainly elderly and immunocompromised patients [1]. Incidence remains low, at around 0.12 to 2.99 cases per 100,000 persons, but an increase in certain countries has been noted [2,3,4,5]. This neoplasm is well known for its aggressive behavior, with high recurrence and low overall survival [1]. It is known to develop mainly in the elderly white population, with a propensity toward male patients [6]. UV exposure and immunosuppression with Merkel cell polyomavirus (MCPyV) are well known risk factors [7,8,9]. In order to improve survival and provide adequate therapies, treatment requires a multidisciplinary and multi-modal approach. Curative treatment relies mainly on wide margin excision of the primary tumor with lymph node assessment, and eventually, adjunctive radiation therapy [10]. Systemic therapies are mainly reserved for distant metastatic disease. Different chemotherapeutic regimens, often platinum-based, are described in small studies with variable benefits, and immunotherapies such as avelumab, pembrolizumab, and nivolumab are currently being investigated [10]. Avelumab is currently recommended as a first line of treatment for metastatic MCC [11]. Lymph node (LN) metastasis has been identified as a strong prognostic factor of disease recurrence and lower survival [12,13,14]. LN biopsy is required to adequately plan oncological management of the patients. Even in patients with clinically negative LN basins, sentinel lymph node biopsy (SLNB) positivity ranges from 23 to 39% [14,15,16,17,18]. In our institution, plastic surgeons maintain a central role in surgical management of malignant cutaneous tumors by conducting wide margin resections, SLNB, and providing tissue coverage with direct closure or local flaps. To offer a tailored surgical approach, a good knowledge of prognostic factors, treatment patterns and outcomes is required.

Our aim was to analyze how demographic, clinicopathologic and treatment patterns influence the choice of lymph node procedures and lymph node positivity. The Survival Epidemiology and End Results (SEER) database was selected, as it is a large, comprehensive database consisting of multiple US cancer registries, representative of the country’s demographics.

## 2. Materials and Methods

Case selection and inclusion/exclusion criteria: Seventeen SEER registries were searched for all MCC cases between 2000 and 2019, using ICD-O3 code 8247/3 [19]. All skin primary sites were selected. Mucosal, subcutaneous, and other MCCs where the primary location was not the skin were excluded, as they are rare occurrences, and their behavior might differ from classical cutaneous forms. Furthermore, cases where the precise localization was not specified were excluded. Cases matching the selection criteria were extracted and processed through IBM SPSS version 28 (IBM, Armonk, NY, USA) for statistical analysis.

Variables of interest were age, sex, surgical therapy on the primary site, number of regional nodes examined, number of positive regional nodes, SEER stage, radiotherapy, chemotherapy and primary site location. We defined the type of LN procedure according to the number of LNs examined. Cases where the number of nodes examined was stated as unknown were excluded from analysis. We arbitrarily defined as SLNB/LN sampling all procedures collecting six or fewer LNs. Procedures collecting more LNs were considered as therapeutic LN dissection. LN positivity was then assessed for cases reported as SLNB/LN sampling, and distribution according to variable was analyzed.

Cases stratification: The age variable was arbitrary divided in 3 categories: less than 65, 65 to 79, and 80 years old and more. This subdivision aimed at differentiating patients that were relatively fit (<65 years) from elderly patients (≥80) where aggressive treatment might not be indicated due to comorbidities. Tumor size was also sub-divided into 4 categories: <1 cm, 1 to 2.9 cm, 3 to 4.9 cm and ≥5 cm. The 1 cm cut-off was defined following the National Comprehensive Cancer Network (NCCN) guidelines, where a primary tumor of more than 1 cm is considered as high risk [10]. We added more arbitrary size divisions to identify how tumor size might influence the positivity rate of LN. Surgery with more or less than 1 cm margin was extracted from the RX Summ—Surg Prim Site (1998+) SEER variable. Stage at diagnosis was analyzed using the SEER summary stage variable, as AJCC stage was reported inconsistently across the cohort [20]. There was only one patient reported as “in situ” in the cohort, and to ease analysis, that patient was included in the localized group corresponding to disease locally limited to the dermis and subcutaneous tissue (AJCC I and IIA equivalent). Regional disease included direct extension into underlying structures (muscle, cartilage, bone) (AJCC IIB) or regional lymph node invasion (AJCC III). Distant disease included all metastatic diseases (AJCC IV).

Statistical analysis: Co-variates were compared using the chi-squared test in univariable analysis. If the value of a variable was stated as unknown, it was not included in statistical analysis. A *p*-value <0.05 was considered statistically significant. Results are presented in a table with row percentages.

## 3. Results

We identified 10,182 cases with MCC, of which 9185 met the inclusion criteria. Mean age was 75.8 years old (σ = 11.4). The cases included 5774 males (62.9%) and 3411 females (37.1%). Mean tumor size was 23.3 mm (σ = 30.9). Number of LNs assessed was reported for 8657 cases, which were divided into 4446 cases without LN procedure, 3139 LN sampling/SLNB, and 1072 therapeutic LN dissections. Status of LN (positive/negative) was reported in 4481 patients, of which 1911 had positive LN (42.6%). The primary site surgery was divided into 1060 cases without surgery (11.7%), 5017 cases with less than 1 cm margin surgery (55.3%), and 2998 cases with margins of 1 cm or more (33%). For 83.1% of the patients who did not receive surgery, the reason was reported as “not recommended”; for 4%, as “recommended, patient refusal”; and for 12,2%, as “recommended, reason unknown”. Of the 3832 patients who received radiotherapy, 3794 (99%) were classified as having adjuvant therapy, 22 as having neo-adjuvant therapy, and 16 as having radiotherapy before and after surgery. Mean overall survival from diagnosis was 47.4 months (median 28 m, σ = 49.7) for the cohort.

When assessing the distribution of LN procedures (Table 1), diagnostic and therapeutic lymphadenectomy tended to decrease with patient age, with 67.6% of patients aged 80 or more receiving no LN procedures. Female patients had fewer therapeutic LN dissections (9.2%). In the head and neck, LN procedures were less frequent, with 64.1% of patients undergoing no LN procedure. When assessing the association between stage at diagnosis and LN procedures, we noted a similar proportion of SLNB/LN sampling between localized and regional disease, whereas therapeutic dissections were mainly performed in the regional group. Distant disease was associated with a low rate of LN procedures. When assessing the relation between LN procedures and tumor size, we observed a trend with an increasing ratio of therapeutic dissection according to tumor size. Patients who did not had primary site surgery did not receive LN procedures (88.5%), and patients with more than 1 cm margins were associated with more LN biopsies or sampling (47.9%) and more therapeutic dissections (17.8%). Use of radiation therapy was associated with more LN procedures (SLNB/LN sampling, 41.8%; and therapeutic LN dissection, 17%). Use of chemotherapy was also associated with more LN procedures (SLNB/LN sampling, 28%; and therapeutic LN dissection, 23.5%).

Increasing age was associated with more positive LNs (Table 2). Positive LNs were noted in 28.8% of males, similar to the share (26.6%) for females. Primary tumor location in the trunk was associated with higher LN positivity (40.8%). Increasing tumor size was correlated with increasing LN positivity. However, the LN positivity rate stopped increasing between tumor sizes of 3 to 4.9 cm and 5 cm or more. Patients who did not receive primary site surgery were associated with up to 91% of LN positivity compared to patients who received surgery (26.9%/25.9%). Of the patients who did not receive any surgery, only 7.7% had a lymph node sampling/SLNB (N = 67), and 3.9% a therapeutic dissection (N = 34). The use of radiation therapy and chemotherapy was associated with higher LN positivity of 35.7% and 65.7% respectively.

## 4. Discussion

This is, to our knowledge, the largest cohort where LN procedures and LN positivity were assessed and compared between demographic, clinicopathologic and treatment patterns for MCC.

When assessing the distribution between lymph node procedures and the different variables analyzed, we noted that increasing age was associated with fewer LN biopsies and therapeutic dissections. However, LN positivity among older patients was higher. A possible explanation for this observation might be that in younger patients, a more aggressive approach to node evaluation was conducted, whereas for elderly patients, only those with higher suspicion of node involvement were assessed, also explaining the higher rate of positive LN. Conic et al. found similar results in a smaller cohort based on the national cancer database with lower SLNB and more positivity of LN with increasing age [21].

Interestingly in this cohort, females had slightly fewer therapeutic LN dissections compared to males while maintaining relatively similar rates of SLNB. The positivity rate of SLNB among females and males did not differ significantly in this cohort. Conic et al. identified similar results in the National Cancer Database (NCD) [21]. While gender does not seem to influence SLNB positivity rate, a small Dutch cohort identified male gender as a strong negative predictor of overall survival [22].

In the head and neck, we noted that SLNB and LN sampling were less frequent than in other sites. Current NCCN guidelines state that SLNB in the head and neck region might not be reliable due to multiple LN basins and suggest adjunctive radiotherapy of the nodal basins [10,23]. That is also the case for the midline trunk [10]. This might explain the lower prevalence of SLNB while maintaining similar therapeutic LN dissection rates. Interestingly, the rate of therapeutic LN dissections was lower in lower limb MCC. While MCC etiology might be linked to sun exposure, Merkel cell polyomavirus and immunosuppression have been identified as important risk factors for oncogenesis in the lower limbs. Our previous study identified that overall survival was better in lower limb MCC than that in other regions, suggesting a possibly different tumor behavior [7]. However, as Schadendorf et al. underlined in their review, the correlation between Merkel cell polyomavirus status and survival is unclear [24]. Current evidence suggests that even if LN drainage basins might differ, SLNB maintains an important role in defining adequate treatment strategy and patient prognosis [13,25,26]. The high LN positivity rates observed in this study (20.6% to 40.8%) suggest the importance of lymph node assessment. Interestingly, we found that upper extremity was associated with a lower LN positivity rate than other regions. Soltani et al., who also compared LN positivity between upper extremity and other regions in the SEER, explained this observation with tumors diagnosed at an earlier stage, with less regional and metastatic invasion [6].

With increasing tumor size, the SLNB rate decreased in favor of increasing therapeutic LN dissection. However, in tumors of more than 5 cm in size, LN procedures rates started to decrease. This can be explained by advanced tumors for which systemic therapy might be proposed, thus not requiring LN dissection. In cases involving SLNB, LN positivity increased strongly with tumor size. Previous studies already identified tumor size as a strong predictor of survival, with increasing size correlated with higher regional or metastatic invasion [22,26,27,28,29,30]. In current NCCN recommendations, a tumor size of 1 cm or more is considered a high-risk tumor requiring multi-modal treatment strategies [10]. Furthermore, tumor size is involved in the eighth AJCC classification of MCC, with a 2 cm cut-off between stage 1 and more advanced stages of disease (stage 2 and higher), and is considered a strong predictor of worse prognosis [31,32].

In patients who did not receive primary site surgery, LN procedures were limited (~10%). Usually, patients who do not receive primary site surgery are not considered for curative intent, as excision with 1 to 2 cm margins is the recommended treatment with adjunctive radiotherapy if the tumor is considered high-risk [10,33,34]. If a surgery with narrow margins is being considered due to structural proximity or morbidity, then adjunctive radiotherapy should be considered for the resection site [10]. The few patients without primary site surgery who had SLNB had a high LN positivity rate (91%), meaning a probable overall worse prognosis. Often, those patients are orientated toward a palliative systemic treatment. In some cases, definitive radiotherapy for the primary site can be considered [10,34,35]. When lesion excision was conducted, the rates of LN diagnostic and curative procedures increased according to surgical margins. This can be explained by more aggressive nodal basin management in patients orientated for curative treatment (>1 cm margins). The SLNB positivity rate was relatively similar between surgery < or ≥1 cm margins.

The use of radiotherapy was associated with more aggressive lymph node basin management, with 41.8% SLNB and 17% therapeutic dissections. The SLNB positivity rate was high, at 35.7%. Radiotherapy maintains a central therapeutic role in MCC for management of the nodal basin as adjuvant therapy or also as the sole therapy [36]. Our results suggest that radiotherapy was mainly given in cases with curative intent because the rate of LN procedures was high. The high rate of LN positivity can be explained by the fact that radiotherapy was used as adjunctive treatment if LNs were positive or at risk of false negativity [10]. However, this variable might be subject to important bias: locations targeted by radiotherapy are not specified in the SEER Database, meaning it is not possible to know if the radiotherapy targeted the primary tumor or the lymph node basin. Furthermore, the use of radiotherapy was reported as yes or no/unknown, meaning there was potentially a high rate of false negatives in the use of radiotherapy.

The use of chemotherapy was associated with a lower rate of SLNB, but higher rate of therapeutic LN dissections. The use of systemic therapy in patients with metastatic disease can explain the low rate of SLNB, as LN sampling is not necessary if disease is already present in distant locations. Chemotherapy was used also in patients who had therapeutic LN dissection. This indication can be explained by the association between the quantity of positive LNs in the dissection and survival, meaning chemotherapy was used in some cases to try to improve survival and control potential distant microscopic disease [12]. Furthermore, this hypothesis is reinforced by the high rate of SLNB positivity (65.7%) in our cohort when chemotherapy was used. Currently, the indications for chemotherapy are limited, as immunotherapy is the recommended systemic treatment in recurrent or disseminated disease [10,11]. Indications for chemotherapy remain to be investigated, as MCC seems to be a chemo-sensitive tumor, with studies reporting variable response rates to diverse drug regimens [37,38]. However, evidence supporting the benefit of chemotherapy to survival is limited [10,37,39,40]. Because immunotherapy is not reported in the SEER database, it was not possible to analyze LN procedures and positivity according to the use of immunotherapy. Furthermore, the chemotherapy variable presented the same limitation as radiotherapy, with a potential bias due to a high rate of false negatives.

Age, primary site location and tumor size are patient and tumor characteristics influencing the rate of SLNB positivity. They are associated with an increasing rate of LN procedures if the intent is curative. However, the results of this study might be influenced by potential bias. Because the intent of LN procedures was not specified for the majority of MCC cases, we decided to set an arbitrary cut-off of 6 LNs assessed between diagnostic and therapeutic dissections, meaning the rate of SLNB/LN sampling and therapeutic dissections might have been under/over-estimated. Furthermore, the SEER database is known to be subject to diverse bias, mainly due to the lack of precision in variables and missing values.

Despite those limitations, to our knowledge, this study provides the largest analysis of LN procedures and SLNB positivity according to demographic, tumor, and treatment characteristics. The results support current NCCN and European guidelines where SLNB is recommended for all tumors with a clinically negative lymph node basin [10,14]. European guidelines recommend ultrasonography of the lymph node basin to confirm cN0 disease [14]. If lymph nodes are clinically positive, the guidelines suggest fine-needle or core biopsy to confirm the presence of regional metastasis [10,14].

In cases of positive SLNB, completion of lymph node dissection is recommended by the NCCN and can be associated with radiation therapy to the nodal basin [10], whereas the European guidelines recommend radiation therapy to the nodal basin in cases of microscopically positive SLNB, eventually associated with complete lymph node dissection [14]. For clinically positive LNs, European guidelines recommend a complete LN dissection and adjuvant radiotherapy [14]. However, all recommendations for positive lymph node basin management are based on limited evidence, and further prospective research is needed.

## 5. Conclusions

LN procedures are frequent (49%) in cases of MCC, and the majority are aimed at detecting nonclinical LN metastases (SLNB). Increasing age, increasing tumor size, and truncal location of the primary tumor were associated with increased rates of positive SLNB. Our results support current NCCN and European guidelines and help identify high-risk groups for LN positivity.

## Figures and Tables

**Table 1:** Lymph node procedure distribution according to demographic, tumor and treatment patterns.

	Overall (n = 8657) N	No Lymph Node Procedure (n = 4446) N (%)	SLNB/LN Sampling (n = 3139) N (%)	Therapeutic LN Dissection (n = 1072) N (%)	*p*-Value
**Age**					<0.05
<65	1421	429 (30.2)	723 (50.9)	269 (18.9)	
65–79	3555	1527 (43)	1538 (43.3)	490 (13.8)	
≥80	3681	2490 (67.6)	878 (23.9)	313 (8.5)	
**Gender**					<0.05
Male	5427	2648 (48.8)	2004 (36.9)	775 (14.3)	
Female	3230	1798 (55.7)	1135 (35.1)	297 (9.2)	
**Primary site**					<0.05
Head and Neck	4081	2616 (64.1)	966 (23.7)	499 (12.2)	
Trunk	869	410 (47.2)	338 (38.9)	121 (13.9)	
Upper limb	2367	866 (36.6)	1175 (49.6)	326 (13.8)	
Lower limb	1340	554 (41.3)	660 (49.3)	126 (9.4)	
**Stage at diagnosis**					<0.05
Localized	4806	2679 (55.7)	1945 (40.5)	182 (3.8)	
Regional	1674	263 (15.7)	763 (45.6)	648 (38.7)	
Distant	514	323 (62.8)	94 (18.3)	97 (18.9)	
**Tumor size**					<0.05
<1 cm	1276	524 (41.1)	631 (49.5)	121 (9.5)	
1 to 2.9 cm	2629	1120 (42.6)	1151 (43.8)	358 (13.6)	
3 to 4.9 cm	771	363 (47.1)	259 (33.6)	149 (19.3)	
≥5 cm	477	253 (53)	139 (29.1)	85 (17.8)	
**Type of primary site surgery**					<0.05
No surgery	875	774 (88.5)	67 (7.7)	34 (3.9)	
<1 cm margin surgery	4803	2629 (54.7)	1665 (34.7)	509 (10.6)	
≥1 cm margin surgery	2914	1000 (34.3)	1396 (47.9)	518 (17.8)	
**Radiotherapy**					<0.05
Yes	4002	1649 (41.2)	1673 (41.8)	680 (17)	
No/unknown	4655	2797 (60.1)	1466 (31.5)	392 (8.4)	
**Chemotherapy**					<0.05
Yes	758	368 (48.5)	212 (28)	178 (23.5)	
No/unknown	7899	4078 (51.6)	2927 (37.1)	894 (11.3)	

SLNB: Sentinel lymph node biopsy; LN: Lymph node. NB: percentages expressed are row percentages. *p*-values represent statistical difference between the LN procedure distributions in each variable category.

**Table 2:** Lymph node positivity in SLNB/LN sampling according to demographic, tumor and treatment patterns.

	Positive Lymph Node(s) N (%)	Negative Lymph Node N (%)	*p*-Value
**Age**			<0.05
<65	174 (24.1)	549 (75.9)	
65–79	393 (25.6)	1145 (74.4)	
≥80	312 (35.5)	566 (64.5)	
**Gender**			0.190
Male	577 (28.8)	1427 (71.2)	
Female	302 (26.6)	833 (73.4)	
**Primary site**			<0.05
Head & Neck	276 (28.6)	690 (71.4)	
Trunk	138 (40.8)	200 (59.2)	
Upper limb	242 (20.6)	933 (79.4)	
Lower limb	223 (33.8)	437 (66.2)	
**Tumor size**			<0.05
<1 cm	90 (14.3)	541 (85.7)	
1 to 2.9 cm	355 (30.8)	796 (69.2)	
3 to 4.9 cm	114 (44)	145 (56)	
≥5 cm	55 (39.6)	84 (60.4)	
**Type of primary site**			<0.05
**Surgery**			
No surgery	61 (91)	6 (9)	
<1 cm margin surgery	448 (26.9)	1217 (73.1)	
≥1 cm margin surgery	361 (25.9)	1035 (74.1)	
**Radiotherapy**			<0.05
Yes	598 (35.7)	1075 (64.3)	
No/unknown	281 (19.2)	1185 (80.8)	
**Chemotherapy**			<0.05
Yes	143 (65.7)	69 (32.5)	
No/unknown	736 (25.1)	2191 (74.9)	

NB: percentages expressed are row percentages. *p*-values represent the statistical difference between LN positivity rates in each variable category.

## Data Availability

Raw data available from the SEER program https://seer.cancer.gov/ (accessed on 12 July 2022).
